# SAMHD1 phosphorylation and cytoplasmic relocalization after human cytomegalovirus infection limits its antiviral activity

**DOI:** 10.1371/journal.ppat.1008855

**Published:** 2020-09-28

**Authors:** Simone De Meo, Valentina Dell’Oste, Rosa Molfetta, Valentina Tassinari, Lavinia Vittoria Lotti, Simone Vespa, Benedetta Pignoloni, Daniela Angela Covino, Laura Fantuzzi, Roberta Bona, Alessandra Zingoni, Ilaria Nardone, Matteo Biolatti, Alessandra Coscia, Rossella Paolini, Monsef Benkirane, Fredrik Edfors, Tatyana Sandalova, Adnane Achour, John Hiscott, Santo Landolfo, Angela Santoni, Cristina Cerboni

**Affiliations:** 1 Department of Molecular Medicine, "Sapienza" University of Rome, Rome, Italy; 2 Department of Public Health and Pediatric Sciences, University of Turin, Turin, Italy; 3 Department of Experimental Medicine, "Sapienza" University of Rome, Rome, Italy; 4 Laboratory of General Pathology, Center of Aging Science and Translational Medicine (CeSI-MeT) and Department of Medical, Oral and Biotechnological Sciences G. d’Annunzio University, Chieti, Italy; 5 National Center for Global Health, Istituto Superiore di Sanità, Rome, Italy; 6 Neonatal Unit, Department of Public Health and Pediatric Sciences, University of Turin, Turin, Italy; 7 Institut de Génétique Humaine, Laboratoire de Virologie Moléculaire, CNRS-Université de Montpellier, Montpellier, France; 8 Science for Life Laboratory, Department of Medicine Solna, Karolinska Institute, and Division of Infectious Diseases, Karolinska University Hospital, Stockholm, Sweden; 9 Istituto Pasteur Italia-Cenci Bolognetti Foundation, Rome, Italy; 10 IRCCS, Neuromed, Pozzilli, Isernia, Italy; Emory Vaccine Center, UNITED STATES

## Abstract

SAMHD1 is a host restriction factor that functions to restrict both retroviruses and DNA viruses, based on its nuclear deoxynucleotide triphosphate (dNTP) hydrolase activity that limits availability of intracellular dNTP pools. In the present study, we demonstrate that SAMHD1 expression was increased following human cytomegalovirus (HCMV) infection, with only a modest effect on infectious virus production. SAMHD1 was rapidly phosphorylated at residue T592 after infection by cellular cyclin-dependent kinases, especially Cdk2, and by the viral kinase pUL97, resulting in a significant fraction of phosho-SAMHD1 being relocalized to the cytoplasm of infected fibroblasts, in association with viral particles and dense bodies. Thus, our findings indicate that HCMV-dependent SAMHD1 cytoplasmic delocalization and inactivation may represent a potential novel mechanism of HCMV evasion from host antiviral restriction activities.

## Author summary

Human cytomegalovirus (HCMV) is among the most widespread viruses worldwide, causing severe systemic illness in individuals without a fully competent immune system (*e*.*g*., transplanted patients and infants). Therefore, host immune response is crucial to control HCMV replication and dissemination throughout the organism. Some frontline proteins belonging to the innate immune system, known as restriction factors, are already expressed and active even before a pathogen infects a cell and are able to mediate a cell-intrinsic resistance to infections. Among them is SAMHD1, a nuclear deoxynucleotide triphosphate (dNTP) hydrolase that limits the availability of intracellular dNTPs, essential components of growing DNA molecules, including those of replicating viruses. We thus investigated SAMHD1 ability to counteract HCMV infection. We show that SAMHD1 expression was upregulated after infection, but with only a modest effect in limiting viral replication. This was mainly due to HCMV-induced phosphorylation at Threonine-592 (the key residue known to negatively regulate viral restriction), to phosphoT592-SAMHD1 relocalization to the cytoplasm of infected cells, and to its association with viral particles. The unprecedented observation of relocation of phosphoT592-SAMHD1 outside the nucleus may affect SAMHD1 function upon HCMV infection, and thus might represent a novel HCMV-mediated immune evasion strategy that will need further investigations.

## Introduction

Human cytomegalovirus (HCMV) is a widely diffused β-herpesvirus infecting most of the population worldwide. Generally, HCMV infection is not associated with disease, but in individuals with an immature or compromised immune system, it can be a serious and even life-threatening pathogen [1]. In fact, HCMV disseminates throughout the body by replicating in a wide variety of cell types, including fibroblasts, epithelial and endothelial cells, macrophages and CD34^+^ progenitors, where it establishes a latent infection [2].

Before adaptive immunity develops, several components of the innate immune system cooperate to limit the initial stages of HCMV replication and dissemination, thus representing the frontline antiviral defense of the host [3]. In particular, host innate restriction factors (RFs) mediate a cell-intrinsic resistance that arrests the viral life cycle at specific steps of infection, replication, and maturation [3]. In relation to HCMV, the best characterized RFs are IFI16, viperin and members of the APOBEC3 family [4].

Sterile α motif (SAM) and histidine-aspartic acid domain (HD)-containing protein 1 (SAMHD1) is a nuclear deoxynucleotide triphosphate (dNTP) hydrolase that regulates the intracellular dNTP pool by catalyzing the hydrolysis of dNTPs into the constituent deoxynucleosides and inorganic triphosphates [5,6]. Through this catabolic activity, and in equilibrium with the biosynthesis of dNTPs controlled by the enzyme ribonucleotide reductase (RNR), the pool of dNTPs is maintained at a level appropriate for cellular DNA replication and repair, but perhaps unsuitable for viral replication [7,8].

SAMHD1 activity is best characterized as a host restriction factor for HIV-1 [9–11], while HIV-2 and some related simian viruses (SIVs) encode the antagonist Vpx, an accessory protein targeting SAMHD1 for ubiquitination and proteasomal degradation [9,10,12]. With respect to DNA viruses, SAMHD1 has been shown to restrict replication of herpes simplex-1 (HSV-1), vaccinia (VACV), hepatitis B (HBV) and Epstein-Barr (EBV) viruses [13–16], as well as early steps of HCMV and MCMV infection [17–19].

SAMHD1 contains a nuclear localization signal that keeps the protein predominantly localized in the nucleus [20,21], while the HD domain—peculiar to all phosphohydrolases [22]—contains the dNTPase active site [5,6,23]. The function of the SAM domain is still poorly understood, although it is one of the most abundant and highly evolutionarily conserved protein-protein interaction motifs [24]. In addition, SAM domains can mediate protein-DNA/RNA interactions as well [25,26], and SAMHD1 has been also proposed as a nucleic acid binding protein with exonuclease activity [27], though this finding is still debated. Lastly, recent evidence has shown that SAMHD1 facilitates homologous recombination of DNA double-stranded breaks, by promoting recruitment of the DNA-end resection factor C-terminal binding protein interacting protein (CtIP) to damaged DNA, thus favoring DNA repair [28].

The critical role of SAMHD1 in nucleotide metabolism and innate immunity is highlighted by the association of SAMHD1 mutations with disease. In particular, mutations affecting its enzymatic activity are associated with Aicardi-Goutières syndrome (AGS), a genetic disorder characterized by a chronic inflammatory response, with increased production of type I interferons and signs of autoimmunity, mimicking a chronic viral infection [29]. Moreover, SAMHD1 is frequently mutated in a variety of cancers, implying a role of this protein in both cellular and viral growth [30–32].

SAMHD1 exists in a monomer-dimer equilibrium, but tetramerizes in the presence of activating nucleotides to form the catalytic competent holoenzyme [5,33,34]. Multiple post-translational modifications regulate SAMHD1 function, although the most extensively studied is the phosphorylation of residue T592 by cyclin-dependent kinases (Cdks) [35–42]. As this phosphorylation is cell cycle-dependent and coincides with increased dNTP levels within the cell, it has been proposed that it may affect the dNTPase activity of SAMHD1, thereby promoting DNA replication [35,38–42]. Unphosphorylated SAMHD1 species are instead predominant in non-cycling G_0_/quiescent cells and correspond to a state of reduced dNTP levels [35,37,39,42].

Besides cell cycle control, mounting evidence has shown how SAMHD1 phosphorylation at T592 residue can destabilize its tetramer conformation, thereby hampering the restriction [34–38,40,43–46]. In fact, only SAMHD1 dephosphorylated at T592 actively restricts HIV-1 [35–38,45], as well as HBV [47,48]. Together, these results demonstrate the crucial importance of phosphorylation as regulatory mechanism able to fine tune and calibrate SAMHD1 activity.

In this study, we addressed the role of SAMHD1 in HCMV infection by investigating its expression levels and phosphorylation status after infection of different cellular models with distinct HCMV strains. We also examined how SAMHD1 affects the capacity of HCMV to replicate and the potential viral evasion mechanisms used to bypass SAMHD1 activity.

## Results

### HCMV infection increases the expression of SAMHD1

**Fig 1:**
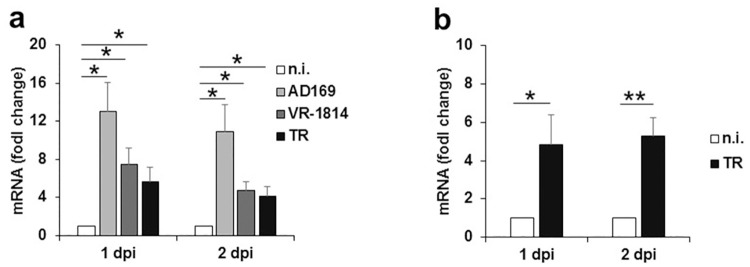
Upregulation of SAMHD1 mRNA in HCMV-infected cells. Primary human foreskin fibroblasts (HFFs) (panel a) or PMA-differentiated THP-1 cells (panel b) were infected with the indicated HCMV strains at an MOI of 1, or not infected (n.i.). At the indicated times after infection, real-time PCR was performed using primers specific for SAMHD1, or for the housekeeping gene GAPDH. Data from three (a) or two (b) independent experiments, expressed as fold change units ± SE, were normalized with GAPDH and referred to n.i. cells considered as calibrators and set ad 1. dpi, day post-infection; *, p < 0.05; **, p < 0.01.

To study the role of SAMHD1 in HCMV infection, we first investigated if its expression was modulated at both mRNA and protein levels in different cell types. SAMHD1 mRNA expression was analyzed in primary human foreskin fibroblasts (HFFs) and macrophage-derived THP-1 cells infected with laboratory or clinical HCMV strains and subjected to reverse transcription-quantitative PCR (RT-qPCR) analysis at day 1 and 2 post-infection (dpi). As shown in Fig 1a, HCMV infection (multiplicity of infection, MOI 1 PFU/ml) of HFFs caused a significant upregulation of SAMHD1 mRNA levels by 1 and 2 dpi, compared to mock-infected HFFs. This effect was most pronounced with the AD169 HCMV strain (i.e., ~13 fold at 1 dpi and ~11 fold at 2dpi), but was also confirmed with two other HCMV strains, VR-1814 and TR, although to a lesser extent (Fig 1a). Notably, SAMHD1 was also upregulated by ~5 fold upon HCMV infection of differentiated THP-1 macrophage-like cells, which are considered a physiologically important target for HCMV infection (Fig 1b) [2].

**Fig 2:**
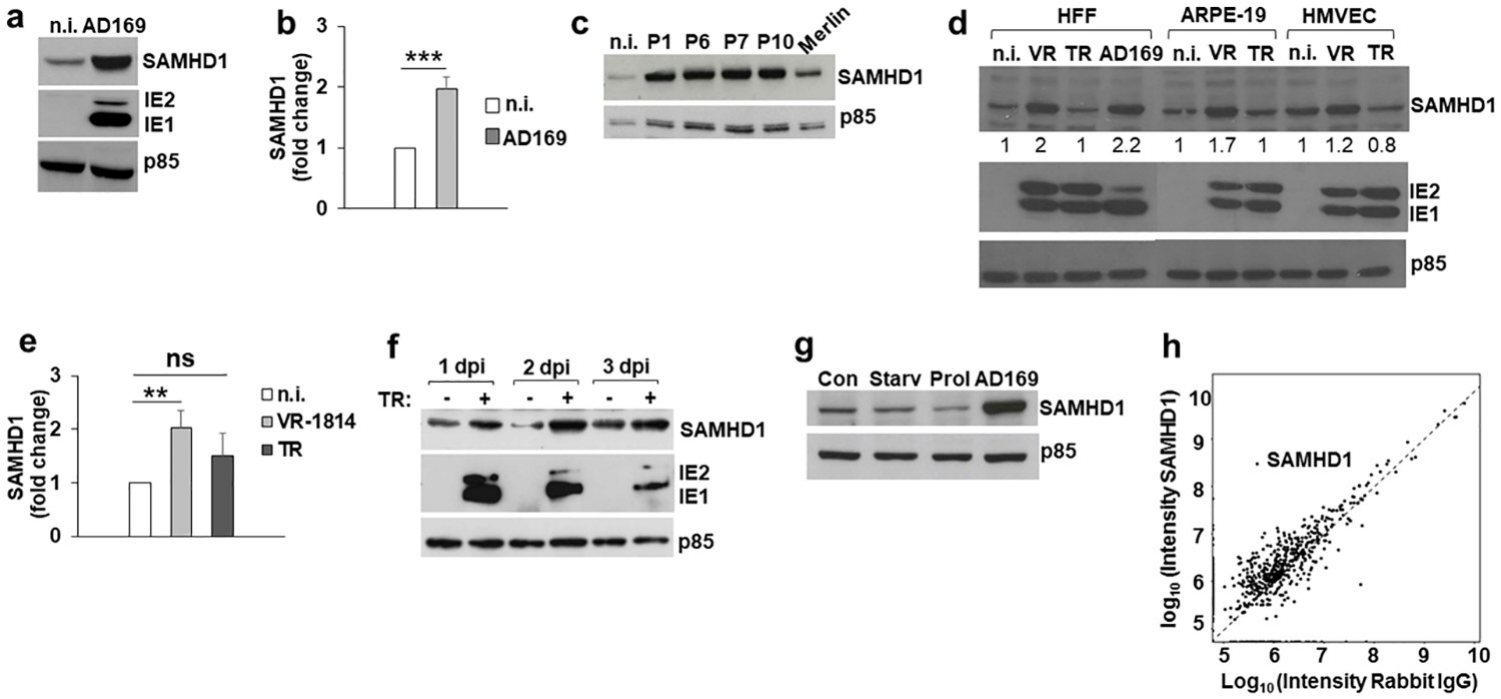
SAMHD1 protein expression in HCMV-infected cells. a) SAMHD1 levels were analyzed by immunoblotting in cell lysates of HFFs not infected (n.i.) or infected with AD169 at an MOI of 1 for 3 days. Expression of IE1/IE2 viral antigens was used as control for infection, while the p85 subunit of PI3K was used as loading control. A representative experiment out of six is shown. b) The relative amount of SAMHD1 protein, normalized to that of p85, was determined by densitometric analysis and is relative to that of n.i. cells, which was arbitrarily set as 1. Data are expressed as mean ± SE of eight independent experiments. c) SAMHD1 expression in HFFs not infected (n.i.) or infected with HCMV clinical isolates collected from infants affected by congenital HCMV infection (P1, P6, P7 and P10), or with Merlin. Data derive from one representative experiment out of two. d) SAMHD1 expression in HFFs, ARPE-19 and HMVEC cells infected with the indicated HCMV strains. Data derive from one representative experiment out of two, performed at the same time with all conditions. Numbers indicate the relative amount of SAMHD1 protein, determined as in panel b). e) The relative amount of SAMHD1 protein from HFFs infected with the indicated clinical strains was determined as in panel b). Data are expressed as mean ± SE of eight independent experiments. All data derive from cells infected at an MOI of 1 and harvested at 3 dpi. f) PMA-differentiated THP-1 cells not infected or infected with TR at an MOI of 1 for the indicated times. Expression of SAMHD1, viral antigens and p85 was evaluated as above. A representative experiment out of two is shown. g) SAMHD1 expression in HFFs induced to reach confluence (Con), serum-starved (Starv), proliferating (Prol), or infected with AD169 at an MOI of 1 for 3 days. Data derive from one representative experiment out of two. h) Antibody validation of the anti-SAMHD1 antibody by immunocapture MS. Proteins enriched by the antibody towards SAMHD1 (*y*-axis) are plotted versus non-specific enrichment by one Rabbit IgG pool (*x-*axis). Enriched proteins identified are quantified by label-free quantification and plotted as intensities (log_10_). **, p < 0.01; ***, p < 0.001; ns, not significant.

Consistent with real-time PCR data, SAMHD1 protein expression increased in HCMV-infected cells, as previously reported (Fig 2) [16,17,49]. In particular, the constitutive expression of SAMHD1 detected in uninfected HFFs was significantly upregulated upon infection with either AD169 (Fig 2a, 2b and 2d), VR-1814 or TR (Fig 2d and 2e), or fresh primary HCMV isolates collected from infants with congenital infection (Fig 2c). SAMHD1 upregulation was observed also in HCMV-infected human adult retinal pigment epithelial cells (ARPE-19) and microvascular endothelial cells (HMVEC) infected with VR-1814 (Fig 2d) and in differentiated THP-1 cells infected with TR strain (Fig 2f). In general, we exclude the possibility of an effect of bystander uninfected cells in the upregulation of SAMHD1, since a high percentage of infected cells was observed in most experimental settings, as detected by intracellular staining with an anti-IE1/IE2 mAb and FACS analysis (Table 1). In time-course experiments performed on HFFs infected with AD169 at an MOI of 1, the increase in SAMHD1 protein levels was detectable at early time-points (8 h-1 dpi) and persisted for up to 4 dpi (Fig 4a).

**Table 1:** Percentage of IE1/IE2+ cells.

	HFF	ARPE-19	HMVEC
AD169	81 ± 2 (10)	*na*	*na*
VR-1814	67 ± 6 (5)	43 ± 2 (2)	33 ± 2 (2)
TR	87 ± 7 (3)	95 ± 1 (2)	82 ± 1 (2)

The different cell types were infected at MOI 1. At 3 dpi, intracellular FACS staining of IE viral antigens was performed with Alexa Fluor 488-conjugated anti-IE1/IE2 mAb. Top numbers indicate the % of IE+ cells ± SE, and bottom numbers the experiments performed. *na*: not applicable, as those combinations result in not productive infection.

Next, we investigated the relative expression of SAMHD1 in HCMV-infected HFFs, compared with proliferating or quiescent fibroblasts, since the latter were described to have higher SAMHD1 levels [8]. To measure the percentage (%) of cells outside the G_0_ phase, an intracellular staining with Ki-67 was also performed, and we confirmed higher SAMHD1 expression in cells that were in cell cycle arrest as a consequence of saturation density or serum starvation (Ki-67^+^ cells: 0%) compared to proliferating cells (Ki-67^+^ cells: 20%); however, the levels of SAMHD1 were much higher upon HCMV infection (Ki-67^+^ cells: 62%) (Fig 2g).

To confirm binding of the correct target and antigen and ensure antibody reproducibility according to the International Working Group for Antibody Validation [50], the anti-SAMHD1 antibody used for Western Blot analysis was validated by immunocapture followed by Mass Spectrometry analysis (MS). SAMHD1 was successfully enriched from HFF cell lysates 641 times versus the negative control (rabbit IgG) (Fig 2h).

Previous studies indicated that type I interferons (IFNs) could play a role in the upregulation of SAMHD1 [51]. To investigate if type I IFN mediated SAMHD1 modulation in our experimental settings, IFN activity was neutralized by adding an anti-IFN receptor (IFNR) blocking mAb to the medium during the infection period (from 1 to 3 dpi). Despite a slight decrease observed at the mRNA level at 1 dpi (S1a Fig), anti-IFNR treatment produced no major changes in SAMHD1 protein levels at 1 and 3 dpi (S1b and S1c Fig), thus excluding the involvement of type I IFNs in SAMHD1 upregulation in our experiments.

Altogether, these results indicate that SAMHD1 is rapidly upregulated at both the mRNA and protein level upon HCMV infection with most viral strain-cell type combinations tested.

### SAMHD1 knockdown has a modest effect on HCMV replication

**Fig 3:**
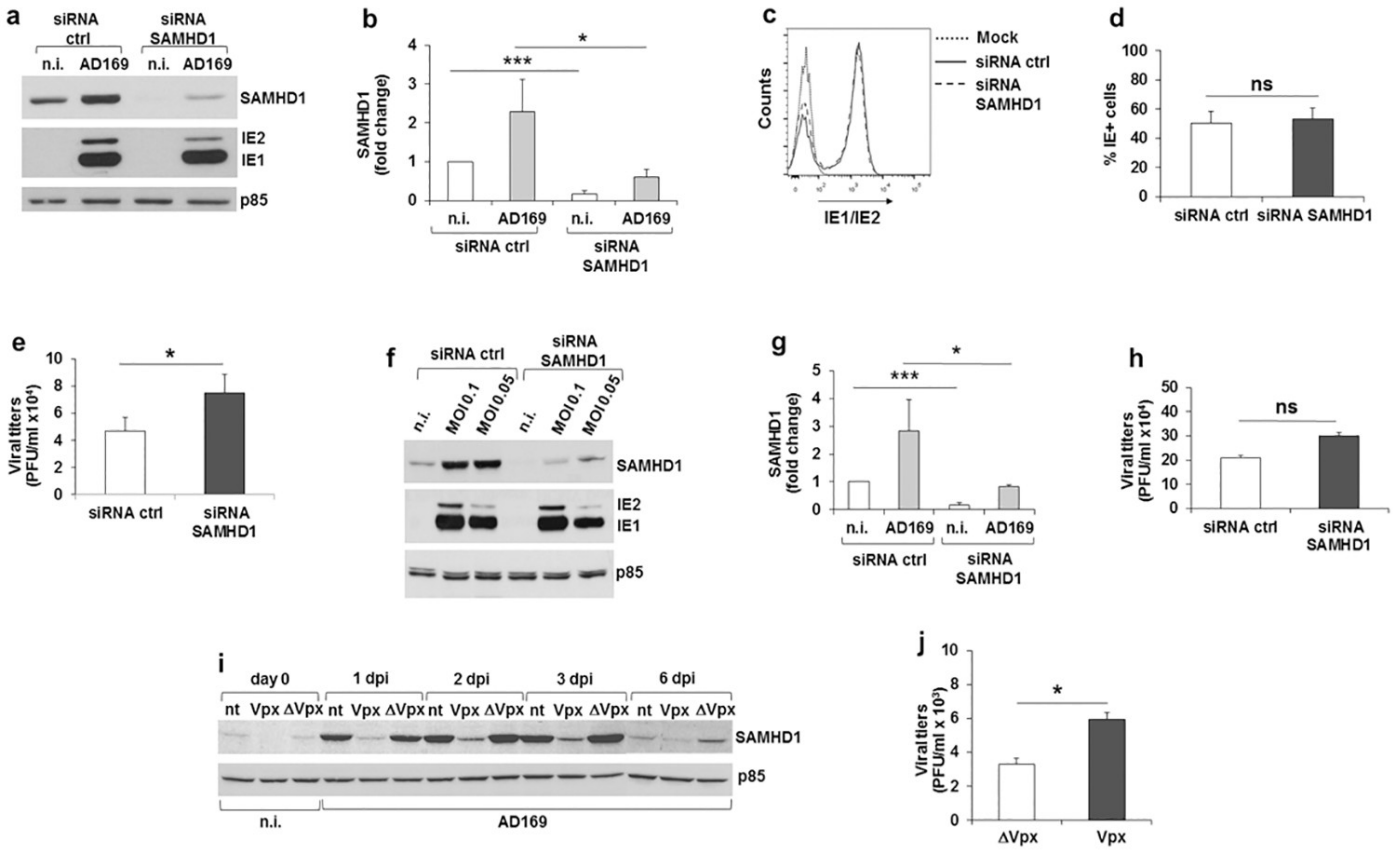
SAMHD1 knock-down by siRNA or Vpx expression and effect on HCMV replication. a-h) HFFs were transfected with SAMHD1 siRNA or non-targeting siRNA (ctrl). Two days later, cells were either infected with AD169 at an MOI of 1 and harvested at 3 dpi (a-e) or infected at an MOI of 0.1 or 0.05 and harvested at 6 dpi (f-h). a) Levels of SAMHD1 and IE1/IE2 viral protein expression were assayed by immunoblotting. The p85 subunit of PI3K was used as loading control. One representative experiment is shown. b) The relative amount of SAMHD1 protein, normalized to that of p85, was determined by densitometric analysis and is relative to that of n.i./siCtrl cells, which was arbitrarily set as 1. Data are expressed as mean ± SE. c) The percentage of IE+ cells was analyzed by FACS after intracellular staining with a specific anti-IE1/IE2 mAb. FACS plots derive from one representative experiment. d) The percentage of IE+ cells is expressed as mean ± SD, as detected by FACS. e) Cell culture supernatants were assayed for infectious virus production by plaque assays. Results are expressed as mean ± SE. All data in panels a-e derive from five independent experiments. f) Immunoblotting at 6 dpi was performed as described in panel a). One representative experiment out of two is shown. g) The relative amount of SAMHD1 protein was determined as in panel b). Data are expressed as mean ± SE of two independent experiments with cells infected at both MOI. h) Viral titers were measured and expressed as in panel e) and derive from cell culture supernatants of two independent experiments, were HFFs were infected at an MOI of 0.1 or MOI of 0.05 for 6 days. i-j) HFFs were infected with single-cycle VLPs loaded with Vpx (Vpx) or unloaded (ΔVpx) at an MOI of 1, and then the same cells were infected with AD169 at an MOI of 1. At different dpi, cells and supernatants were harvested and subjected to immunoblotting and plaque assays, respectively. i) Immunoblotting of SAMHD1 protein expression. One representative experiment out of three is shown. j) Cell culture supernatants were assayed for infectious virus production by plaque assays. Results are expressed as mean ± SE of four experiments at 2–3 dpi. nt, not treated; n.i., not infected cells; *, p < 0.05; ***, p < 0.001; ns, not significant.

To assess whether SAMHD1 acted as a restriction factor for HCMV, we inhibited SAMHD1 expression using two different approaches, small interfering RNA (siRNA) or expression of Vpx, an HIV-2/SIV accessory protein known to degrade SAMHD1 via the proteasome pathway [9,10,12]. First, HFFs were transiently transfected with either a specific siRNA (siSAMHD1) or a non-targeting siRNA as control; after 2 days, cells were infected with HCMV at an MOI of 1, and at 3 dpi both IE expression and progeny virus production were monitored (Fig 3). SAMHD1 silencing was confirmed by immunoblot analysis (Fig 3a), with a significant knockdown efficiency (~70%) among the different experiments (Fig 3b). Although we observed a slight but reproducible reduction of IE2 protein levels in siSAMHD1 cells (Fig 3a), the percentage of IE^+^ HFFs did not vary between siCtrl and siSAMHD1-treated cells, as detected by FACS (Fig 3c and 3d). Rather, HCMV growth was increased by ~1.6-fold in SAMHD1-depleted cells, as measured by plaque assays (Fig 3e).

To determine whether SAMHD1 restriction activity was dependent on the amount of viral input, we assessed the effect of SAMHD1 silencing on HCMV growth in cells infected with lower MOIs (0.1 or 0.05) and at a later time point of infection (6 dpi). Also under these conditions, SAMHD1 silencing had a modest effect on HCMV replication (Fig 3f–3h), with an increase in virus yields of ~1.4-fold, compared to siCtrl cells (Fig 3h).

To confirm these results using a parallel approach, SAMHD1 protein levels were depleted by infecting HFFs with SIV viral like particles (VLPs) loaded with Vpx, or without Vpx (ΔVpx), and then infected with HCMV (MOI 1) (Fig 3i and 3j). SAMHD1 expression was increased after AD169 infection at 1 dpi and persisted up to 6 dpi, whereas SAMHD1 upregulation by HCMV was severely affected in Vpx- but not ΔVpx-expressing cells (Fig 3i). Similar to the results obtained with siRNA, depletion of SAMHD1 by Vpx resulted in a ~2-fold increase of viral titers at MOI 1 at 2–3 dpi (Fig 3j), which was statistically significant compared to ΔVpx VLPs. No major differences were observed in SAMHD1 expression levels or viral titers after infection with VLPs at an MOI of 2 and/or by adding HCMV 24 h later. Thus, SAMHD1 displayed a modest antiviral restriction activity against HCMV.

### SAMHD1 is phosphorylated at T592 in HCMV-infected cells

**Fig 4:**
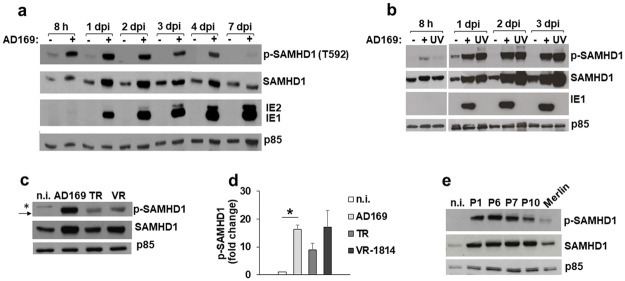
SAMHD1 is phosphorylated at T592 in HCMV-infected cells. a) SAMHD1 phosphorylation at T592 (pT592) was analyzed by immunoblotting with a specific antibody in cell lysates of HFFs not infected or infected with AD169 at an MOI of 1 for the indicated time points. Expression of IE1/IE2 viral antigens was used as control for infection, while p85 was the loading control. A representative experiment out of six is shown. b) pT592-SAMHD1 was analyzed as in panel a), in parallel with an UVB-inactivated AD169 (UV), for the indicated time points. A representative experiment out of two is shown. c) pT592-SAMHD1 was analyzed as in panel a), in HFFs not infected or infected with the indicated strains (MOI 1, 3 dpi). A representative experiment out of three is shown. d) The relative amount of pT592-SAMHD1, normalized to that of p85, was determined by densitometric analysis and is relative to that of n.i. cells, which was arbitrarily set as 1. Data are expressed as mean ± SE of at least three independent experiments. *, p < 0.05. e) pT592-SAMHD1 levels in HFFs infected with HCMV clinical isolates (P1, P6, P7 and P10) or with Merlin. Data derive from one representative experiment out of two. *, unspecific band; black arrow, pT592-SAMHD1 specific band.

Several studies have shown that phosphorylation at T592 negatively regulates SAMHD1 restriction activity [34–38,40,43–48]. The crystal structure of the human SAMHD1 tetramer revealed that residue T592 is localized between two small helices within the C-terminal region of SAMHD1, close to the dimer/dimer interface. Although it is known that this region is critical for the formation of active tetramers, impact of T592 phosphorylation on tetramer formation is still a matter of debate [33–34,43–46,52–53]. Nevertheless, we hypothesized that despite the significant increase in SAMHD1 expression levels detected upon HCMV infection, its modest antiviral activity could be due to inactivation by T592 phosphorylation. Indeed, we observed an upregulation of T592 phosphorylation (pT592) in HCMV-infected HFFs as early as 8 hpi, and pT592-SAMHD1 remained elevated above basal level for up to 4 dpi (Fig 4a). To test if the HCMV-triggered SAMHD1 phosphorylation was due to *de novo* viral protein expression, HFFs were infected with an UVB-inactivated AD169 (UVB-AD169) that does not affect cell entry but blocks gene expression and replication [54]. Interestingly, despite a slight decrease observed at 8 hpi, UVB-AD169 resulted in similar pT592-SAMHD1 levels compared to wild-type virus from 1 to 3 dpi, indicating that phosphorylation events also occurred independently of viral gene expression (Fig 4b).

Likewise, infection of HFFs with TR, VR-1814 or with fresh primary clinical isolates collected from infants led to increased levels of pT592 (Fig 4c–4e), although the levels varied depending on the viral strain, suggesting that this post-translational modification might also be relevant to HCMV pathogenesis.

**Fig 5:**
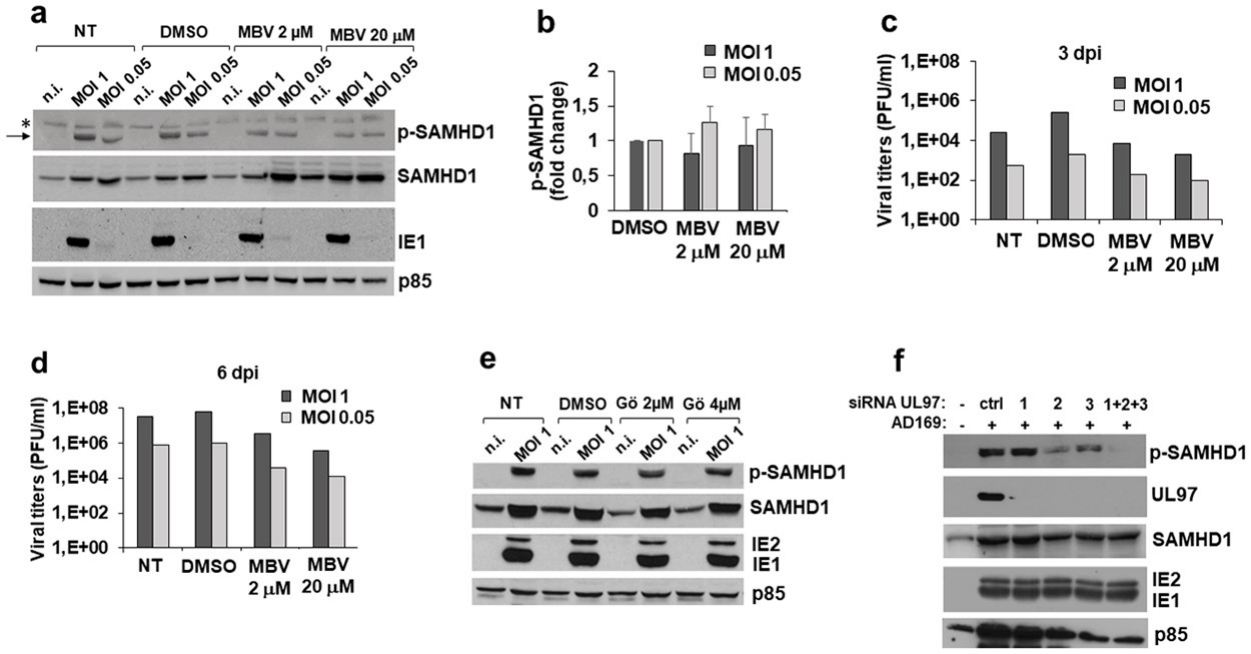
Effects of HCMV kinase pUL97 inhibition on SAMHD1 phosphorylation at T592. a) HFFs were not infected (n.i.) or infected with AD169 at the indicated MOI, in the presence of different concentrations of the pUL97 inhibitor Maribavir (MBV). After removing virus inoculum, MBV was newly added and kept until cell harvesting at 1 dpi. Total or pT592-SAMHD1 levels, expression of IE1/IE2 viral antigens and of p85 were analyzed by immunoblotting as above. A representative experiment out of two performed at 8 hpi, 1, 3 and 6 dpi is shown. b) The relative amount of pT592-SAMHD1 normalized to that of p85 was determined by densitometric analysis and is relative to that of DMSO-treated cells, which was arbitrarily set as 1. Data are expressed as mean ± SE of two independent experiments (MOI 1, 1 dpi). The analysis revealed no statistically significant difference. c-d) Effect of MBV on infectious virus production as measured by standard plaque assays on cell culture supernatants at 3 dpi (c) or 6 dpi) (d). Results derive from one representative experiment out of two. e) Total or pT592-SAMHD1 was analyzed as in panel a) in cells treated with the inhibitor Gö6976 (Gö). A representative experiment out of two performed at 3 dpi is shown. f) HFFs were transfected with three different UL97 siRNAs, alone (1, 2, 3) or in combination (1+2+3), or with a non-targeting siRNA (ctrl). The day after, cells were infected with AD169 at an MOI of 1 and harvested at 3 dpi. Immunoblotting was performed as above. One representative experiment out of two is shown. NT, not treated cells; DMSO, cells treated with the vehicle DMSO; *, unspecific band; black arrow, pT592-SAMHD1 specific band.

Next, we studied the involvement of viral and cellular kinase(s) in the HCMV-dependent T592 phosphorylation. Since T592 residue is phosphorylated at early times post-infection (Fig 4a), we examined whether the HCMV protein pUL97 was involved. pUL97 is a serine-threonine kinase that phosphorylates itself and multiple viral and host proteins, including SAMHD1 as shown recently [16–18, 55–58]. In kinetics experiments, HFFs were infected (MOI 1 or MOI 0.05) in the presence of the pUL97 inhibitor L-benzimidazole riboside maribavir (MBV) [59–61]. As shown in Fig 5 (panels a-b), MBV failed to inhibit T592 phosphorylation at 1 dpi or at later time points, independent of the amount of virus or drug concentration. As a readout of MBV efficacy we analyzed virus growth and, as previously reported [61], MBV had a strong inhibitory effect; at 2 μM, MBV suppressed HCMV replication by ~35- and ~19-fold at days 3 and 6, respectively (at MOI 1), while at higher doses (20 μM) MBV further increased the inhibitory effect by one log magnitude (Fig 5c and 5d). Similarly, treatment with Gö6976, another pUL97 kinase inhibitor [62], did not affect pT592 levels (Fig 5e).

To further investigate the role of pUL97 in T592 phosphorylation with a different approach, HFFs were transfected with a single or a mixture of three different UL97 siRNAs (siRNA UL97). One day after silencing, cells were infected with HCMV for 3 days and pUL97 expression was evaluated. As shown in Fig 5f, silencing by the mixture of three siRNAs had the strongest impact on pT592-SAMHD1 as well as on pUL97 expression, although single siRNAs also significantly inhibited pUL97 protein expression. These observations, together with the results obtained with the pUL97 inhibitors and AD169-UVB, indicate there are multiple factors involved in SAMHD1 phosphorylation and this effect cannot be solely ascribed to pUL97 viral kinase, but may reflect a combinatorial effect of both viral and cellular enzymes.

**Fig 6:**
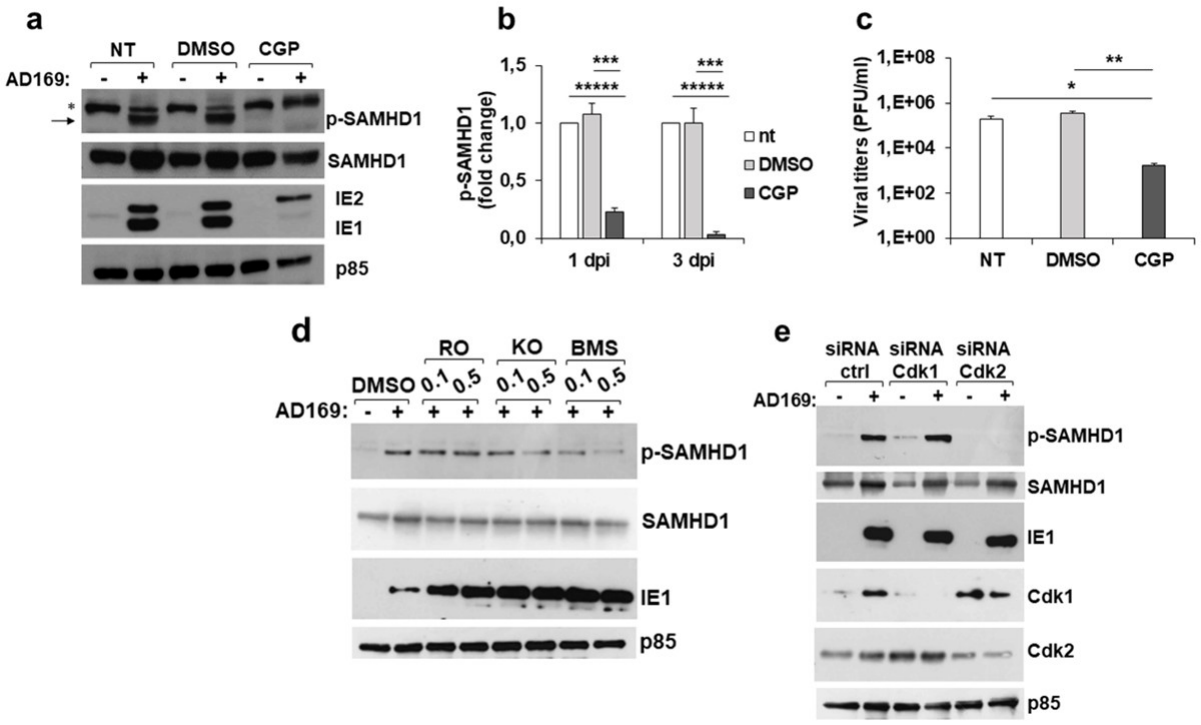
Inhibition of Cdk2 abrogates T592 phosphorylation. a) HFFs were pretreated for 5 h with the Cdk1 inhibitor CGP74514A (CGP), and then not infected or infected with AD169, in the presence of CGP. After removing virus inoculum, the inhibitor was newly added and kept until cell harvesting, at 3 dpi. Total or pT592-SAMHD1 levels were analyzed by immunoblotting, together with IE1/IE2 and p85 expression. A representative experiment out of five is shown. b) The relative amount of pT592-SAMHD1, normalized to that of p85, was determined by densitometric analysis and is relative to that of n.i. cells, which was arbitrarily set as 1. Data are expressed as mean ± SE of four independent experiments performed at 1 and 3 dpi. c) Effect of CGP on infectious virus production as measured by standard plaque assays on cell culture supernatants at 3 dpi. Results derive from seven experiments and are expressed as mean ± SE. d) HFFs were pretreated as in panel a) with the Cdk inhibitors RO-3306 (RO), KO-3861 (KO) or BMS-265246 (BMS) at the indicated concentrations (μM), and then not infected or infected with AD169, in the presence of the inhibitors. After removing virus inoculum, the drugs were newly added and kept until cell harvesting, at 1 dpi. Immunoblotting was performed as above. One representative experiment out of two is shown. e) HFFs were transfected with Cdk1 or Cdk2 siRNA or non-targeting siRNA (ctrl). The day after, cells were not infected or infected with AD169 at an MOI of 1 and harvested at 1 dpi. Immunoblotting was performed as above. One representative experiment out of two is shown. NT, not treated cells; DMSO, cells treated with the vehicle DMSO; *, unspecific band; black arrow, pT592-SAMHD1 specific band. *, p < 0.05; **, p < 0.01; ***, p < 0.001; ****, p < 0.0001; *****, p< 0.00001.

Therefore, to gain further insights into HCMV-induced SAMHD1 phosphorylation, we next asked whether Cdk1/2 were involved in T592 phosphorylation in response to HCMV infection, since cellular cyclin A2/Cdk1/2 complexes have been shown to phosphorylate SAMHD1 at T592 [35–42]. Using either pharmacological inhibition or genetic silencing of Cdk1 and Cdk2, we next identified a preferential requirement for Cdk2-dependent phosphorylation. Two different approaches were used: i) the use of Cdk inhibitors, and ii) Cdk1 or Cdk2 siRNAs. Treatment of HCMV-infected HFFs with the Cdk inhibitor CGP74514A (4 μM) completely abolished T592 phosphorylation at all time points analyzed, from 8 hpi to 6 dpi (Fig 6a and 6b). CGP74514A treatment also dramatically reduced viral replication by ~300-fold compared to untreated controls, as measured by viral titers at 3 dpi (Fig 6c). Similarly, expression of viral IE1/IE2 antigens was also affected (Fig 6a). With the use of other specific Cdk1/2 inhibitors (i.e., RO-3306 and KO-3861, specific for Cdk1 or Cdk2 respectively, and BMS-265246 affecting both Cdk1 and Cdk2), we observed that inhibition of Cdk2 had a major impact on pT592 phosphorylation (Fig 6d). Consistent with this observation, T592 phosphorylation was abolished when Cdk2 was silenced with siRNA (Fig 6e).

Taken together, these results indicate that both the viral protein kinase pUL97 and the cellular kinase Cdk2 were involved in SAMHD1 T592 phosphorylation during HCMV infection.

### Re-localization of SAMHD1 and its association with HCMV particles

We next evaluated the impact of HCMV infection on SAMHD1 localization, with the rationale that the inability of SAMHD1 to restrict HCMV replication may relate to a viral evasion mechanism that altered the nuclear localization of SAMHD1, which contains a classic nuclear localization signal (NLS).

**Fig 7:**
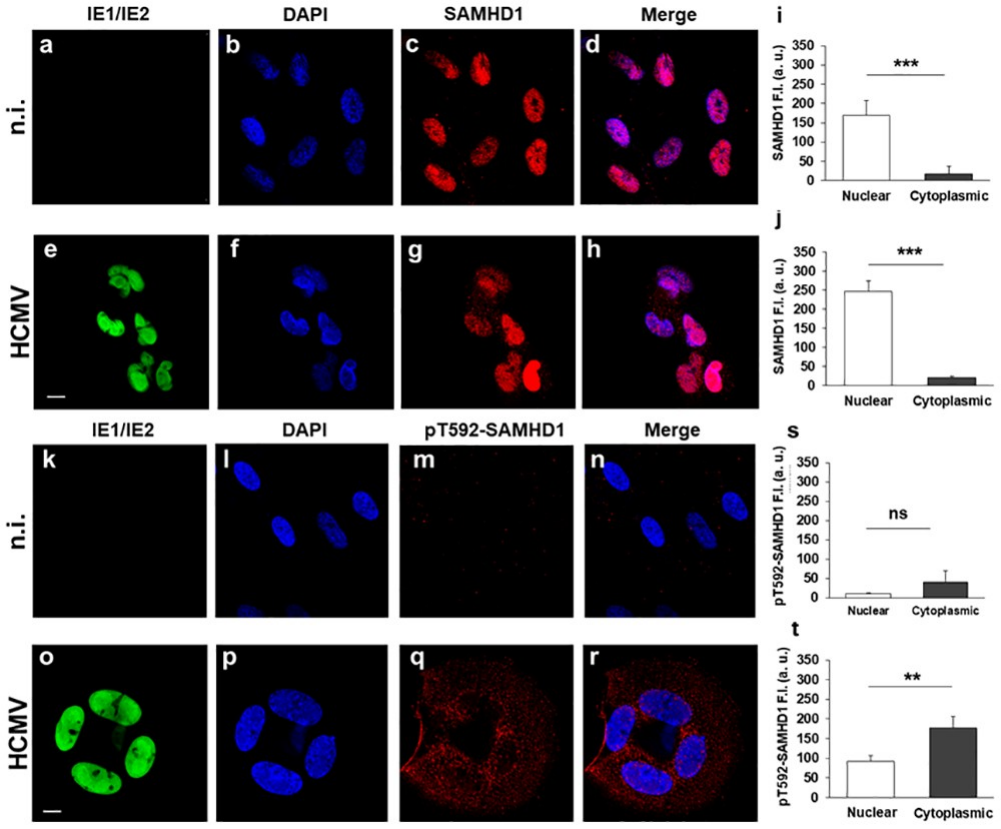
SAMHD1 intracellular localization in uninfected and HCMV-infected cells. HFFs were not infected (n.i.) or infected with AD169 at an MOI of 1 for 3 days. Stainings were then performed using primary antibodies directed against total (c and g) or pT592-SAMHD1 (m and q), followed by Alexa-Fluor 594 conjugated goat anti-rabbit (red), together with IE1/IE2 viral antigens followed by Alexa-Fluor 488 conjugated goat anti-mouse (a, e, k, o) (green) antibodies. Nuclei were stained with DAPI (b, f, l, p) (blue). Confocal images were acquired with a zoom 2 and are shown as single optical slice from one representative experiment out of six. An overlay of blue/red images (d, h, n, r) is also shown. Scale bar: 10μm. Fluorescence intensity (F.I.) of SAMHD1 (i-j) and p-T592 SAMHD1 staining (s-t) was calculated in the nucleus and cytoplasm of 80 cells randomly acquired from 3 independent experiments, as described in materials and methods. Histograms represent the mean ± SE. Displayed values are millions of a.u. ns, not statistically significant difference; ** p < 0.01; *** p < 0.001.

To investigate the subcellular localization of total and pT592-SAMHD1 in uninfected and HCMV-infected cells, confocal microscopy, biochemical fractionation of nuclear/cytoplasmic extracts, and cryo-immunoelectron microscopy (Cryo-IEM) were used. Confocal microscopy revealed SAMHD1 to be constitutively expressed in HFFs (Fig 7c), predominantly in its T592 unphosphorylated conformation (Fig 7m). As expected, HCMV infection upregulated the expression of both unphosphorylated- and pT592-SAMHD1 (Fig 7g and 7q). Remarkably, pT592-SAMHD1 species were found to be mainly localized in the cytoplasm of infected cells (Fig 7p, 7q and 7r), whereas IE proteins retained their nuclear localization (Fig 7e and 7o). To obtain a more exhaustive overview, quantitative analysis of the intensity of SAMHD1 fluorescent signal in the nucleus and cytoplasm was evaluated on about 80 cells from three independent experiments (Fig 7i, 7j, 7s and 7t). This analysis confirmed that pT592-SAMHD1 was predominantly localized in the cytoplasm of infected cells (Fig 7j and 7t). Both anti-SAMHD1 antibodies resulted in a specific binding, as there was a significant reduction in staining intensities upon depletion of SAMHD1 by siRNA (S2 Fig).

**Fig 8:**
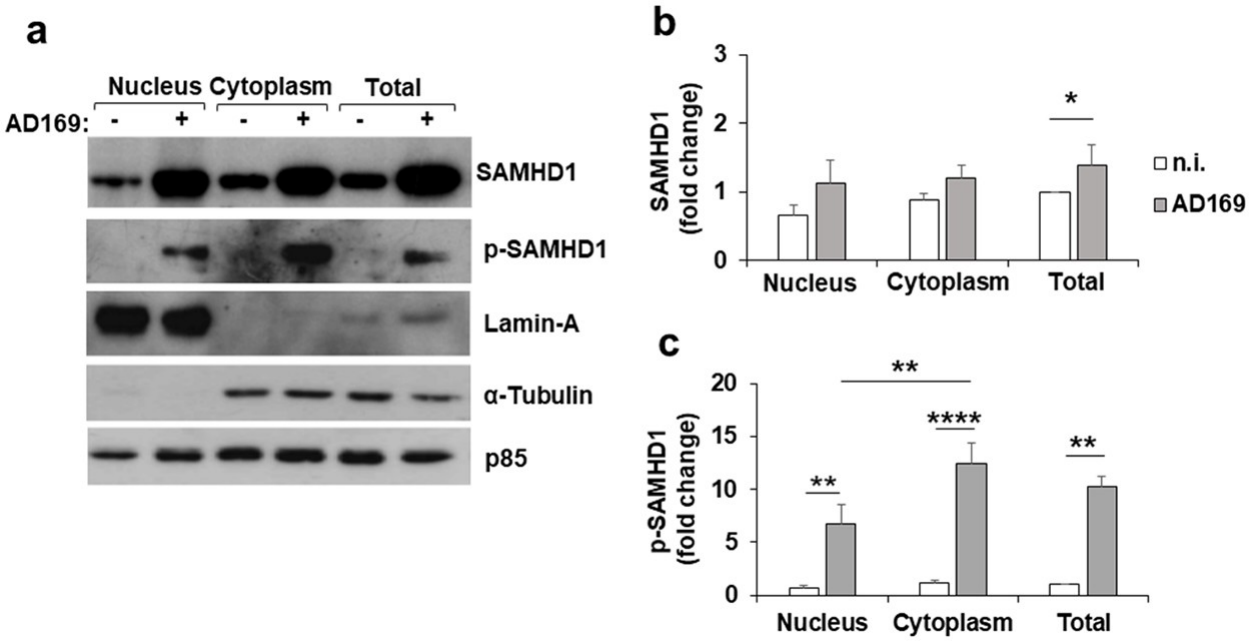
pT592-SAMHD1 is largely localized in the cytoplasm upon HCMV infection. a) HFFs were infected with HCMV at an MOI of 1 and nuclear, cytoplasmic, and total cell extracts were prepared at 3 dpi. Thirty μg of proteins were loaded and subjected to immunoblotting with the indicated antibodies. Lamin-A and α-tubulin were used to confirm fractionation efficiency, and p85 as loading control. Results derive from one representative experiment out of four. b-c) The relative amount of total (b) or pT592-SAMHD1 (c), normalized to that of p85, was determined by densitometric analysis and is relative to that of total cell extracts in n.i. cells, which was arbitrarily set as 1. Data are expressed as mean ± SE of four independent experiments. *, p < 0.05; **, p < 0.01; ***, p < 0.001; ****, p < 0.0001.

To further evaluate pT592-SAMHD1 localization in the cytoplasm, nuclear and cytoplasmic fractionation was performed and the purity of the two fractions was confirmed by immunoblotting with anti-lamin A and anti-tubulin antibodies (Fig 8). The nuclear localization of SAMHD1 was confirmed in uninfected cells, with a fraction of SAMHD1 detectable also in the cytoplasm. After infection, a reproducible increase in SAMHD1 was seen in total cell lysates, with a similar trend in the two cell fractions (Fig 8a and 8b). Consistent with the immunofluorescence results shown in Fig 7, there was a significant amount of pT592-SAMHD1 localized in the cytoplasm of infected cells (Fig 8a and 8c). Collectively, these results demonstrate that a large fraction of pT592-SAMHD1 is localized to the cytoplasm after HCMV infection.

**Fig 9:**
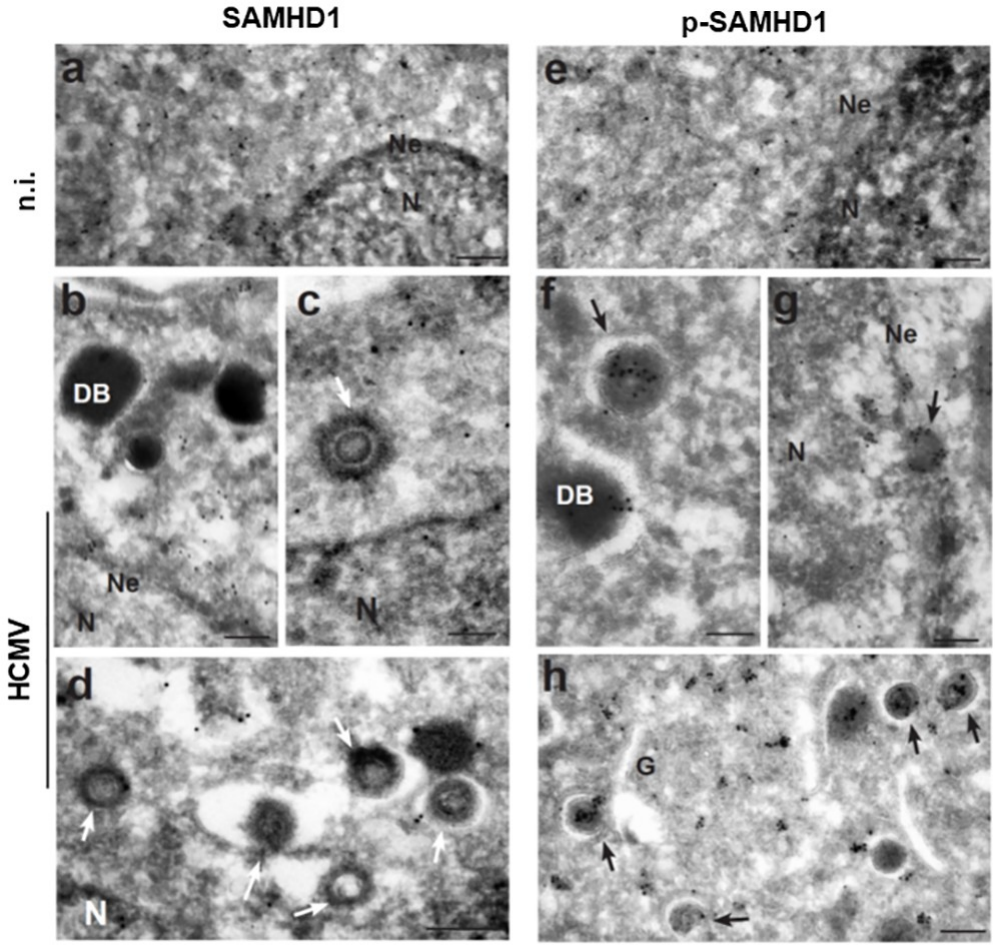
pT592-SAMHD1 associates with viral particles and dense bodies. HFFs, not infected (n.i.) or infected with AD169, were processed at 3 dpi for Cryo-IEM analysis and immunolabeled using primary specific antibodies against SAMHD1 (a-d) or pT592-SAMHD1 (e-h). One representative experiment out of seven different immune gold-labeling experiments is shown. Sparse labeling of SAMHD1 and pT592-SAMHD1 was detected in uninfected cells in the nucleus and in the cytoplasm (a, e). In infected cells, viral structures were not labeled with SAMHD1 gold (b-d; white arrows) but were gold-decorated with pT592-SAMHD1 (f-h; black arrows). N, nucleus; Ne, nuclear envelope; G, Golgi apparatus, DB, dense bodies. Bars a, b, d, e, g, h = 200nm; c, f = 100 nm.

This result prompted us to test whether pT592-SAMHD1 translocation to the cytoplasm was due to its association with viral structures. Ultrastructural analysis of SAMHD1 distribution by cryo-immunoelectron microscopy (Cryo-IEM) failed to detect total SAMHD1 protein association with viral structures in HCMV-infected HFFs (Fig 9c and 9d, white arrows). In contrast, pT592-SAMHD1 colocalized with infectious viral particles, as well as non-infectious dense bodies (Fig 9f and 9h, black arrows). Specifically, the average number of gold particles *per* virion or dense body detected using the anti-pT592-SAMHD1 or the anti-SAMHD1 antibody was 3.20 ± 0.20 (mean ± SEM) and 0.98 ± 0.09 (mean ± SEM), respectively (Table 2).

**Table 2:** Immunogold quantification of total and pT592-SAMHD1 on virions and dense bodies in infected cells (MOI 1).

	SAMHD1 (total) (n = 184)	p-T592-SAMHD1 (n = 184)	Secondary Ab (n = 34)
Mean ± SE (gold/virions+ dense bodies)	0.98 ± 0.09	3.20 ± 0.20	0.65 ± 0,15
*P* value	p > 0.05 (ns)	p < 0.0001 (****)	

Statistical analysis was performed on 184 samples of virions and dense bodies with a Student’s t-test between gold particles counted using anti-SAMHD1 and anti-pT592 antibodies separately, compared to the secondary antibody alone. P-values less than 0.05 were considered statistically significant.

To characterize HCMV proteins binding to SAMHD1 prior to or after phosphorylation, pull-down assays were performed using anti-SAMHD1 and anti-pT592-SAMHD1 antibodies with lysates from HFF infected cells. A comparison of the pull-down assays with the two antibodies demonstrated that the levels of pp65, TRS1, VPAP and IRS1 viral proteins were enriched upon SAMHD1 pull-down, while the amount of IR11 was significantly enriched when anti-pT592-SAMHD1 was used (S3 Fig).

In summary, these results identify for the first time a cytoplasmic localization of pT592-SAMHD1 after HCMV infection in association with viral particles and dense bodies, and the association of this restriction factor with HCMV proteins.

## Discussion

SAMHD1 functions as a central regulator of dNTP metabolism thanks to its ability to hydrolyze dNTPs into deoxynucleosides and inorganic triphosphates, thereby regulating dNTP availability during critical physiological processes such as DNA replication and repair [5,6]. Given that the dNTP pool is essential for viral replication as well, SAMHD1 emerged also as a host restriction factor for retroviruses [9–11,63] and DNA viruses [13–16]. Furthermore, while this manuscript was in preparation, two other groups reported that SAMHD1 can also have a restriction activity against HCMV [17,18]. However, their partially contrasting results implied that additional studies were needed to fully understand the antiviral function of SAMHD1.

In our study, we show that SAMHD1 mRNA and protein levels significantly increase in response to infection with most of the HCMV strains tested–i.e., AD169, VR-1814 and viral isolates from congenitally infected infants–and in different cell types–i.e., primary fibroblasts, epithelial cells and differentiated THP-1 cells. On the other hand, TR was the weakest strain (despite the different cell types were 80–90% infected), and HMVEC the cells in which SAMHD1 modulation was less evident. In relation to recently published studies, while our results are in line with SAMHD1 being upregulated in fibroblasts and in THP-1 cells infected with HCMV [17], they are in contrast with the reduced expression observed by Businger *et al*. in primary HCMV-infected macrophages [18], suggesting that HCMV-mediated regulation of SAMHD1 can be cell type-specific, as well as strain-dependent. Conversely, it appeared to be type I IFN-independent, as no major SAMHD1 modulation was observed upon IFN neutralization by a blocking anti-IFNR mAb.

We also show that the HCMV-dependent upregulation of SAMHD1 protein expression levels is much higher than that observed in cells at any stage of the cell cycle. This observation is somewhat surprising since SAMHD1 is more abundantly expressed in quiescent (G_0_-phase) *vs*. proliferating (S-phase) cells [8,64], and that HCMV is known to recruit cells from G_0_ into G_1_/S phase, and then to arrest cell cycling at the G_2_/M phase [65]. However, a consensus on SAMHD1 expression during cell cycle has not been forthcoming, as other studies demonstrated high expression in proliferating cells [10,11] or no modulation [39,42].

To add a layer of complexity, our data indicate that the antiviral activity of SAMHD1 is not strictly correlated with its fluctuating expression levels; despite upregulation in HCMV-infected cells, SAMHD1 had only a modest capacity to restrict HCMV replication. In fact, SAMHD1 knockdown using specific siRNA or Vpx-expressing VLPs only resulted in an average of ~1.7-fold increase in viral titers, with no major differences in the percentage of cells expressing IE proteins.

Kim *et al*. recently proposed that SAMHD1 exerts its antiviral activity by limiting NF-kB activation and IE expression, thus acting at early steps of viral replication [17]. Although we did not investigate the NF-kB pathway directly, we did not observe an effect of SAMHD1 on IE protein expression; rather we observed a slight but reproducible decrease in IE2 expression in SAMHD1-silenced cells. This change was not confirmed by FACS analysis, potentially because the anti-IE antibody recognizes both IE1 and IE2 proteins. Overall, we did not detect a clear modulation of viral replication following SAMHD1 depletion by either analyzing IE expression or measuring infectious viral particle production.

Phosphorylation of T592 is considered a crucial post-translational modification that negatively regulates SAMHD1 restriction activity [34–38,44–45]. In this regard, we observed a dramatic increase of T592 phosphorylation at early times after infection. A good viral candidate to mediate phosphorylation was the kinase pUL97 which has been shown to phosphorylate itself and multiple viral and host proteins [55–58], including SAMHD1 [16–18]. In our hands, pUL97 inhibitors MBV and Gö6976 had little effect on pT592-SAMHD1 levels, whereas siRNA mediated knock-down of pUL97 generated a strong inhibition of SAMHD1 phosphorylation. Therefore, based on our current results, as well as recently published studies [16–18], we conclude that pUL97 viral kinase is in part responsible for SAMHD1 phosphorylation, although other factors including cellular enzymes could likewise be involved.

Indeed, the results obtained using different Cdk inhibitors and specific Cdk1 and Cdk2 siRNA, showed a major involvement of Cdk2 in pT592 phosphorylation. Our findings are therefore in good agreement with previous observations showing that cellular Cdk1/2 are implicated in phosphorylation of SAMHD1 upon HCMV [16,18], as well as HIV-1 and HSV-1 infections [35–42,66,67]. An additional interpretation of these results is that Cdks promote HCMV replication [65,68] also through pT592-dependent inactivation of SAMHD1, as shown previously for HIV-1 [35,37,38]. Thus, there appears to be a strict interplay between cellular and viral kinases in SAMHD1 phosphorylation, that could be differently regulated depending on the time post-infection.

The absence of significant changes in IE protein expression and/or infectious virus production in cells depleted of SAMHD1 prompted us to address whether T592 phosphorylation could be part of a novel immunoevasion mechanism attributed to HCMV. In particular, we asked whether pT592-SAMHD1 displayed a cytoplasmic delocalization, as described for other nuclear restriction factors [69]. Strikingly, our results show that HCMV-infected cells contain a significant fraction of pT592-SAMHD1 in the cytoplasm, with a cytoplasmic: nuclear ratio of 2:1. This is the first detection of pT592-SAMHD1 outside the nucleus of infected cells, and suggests that relocalization of SAMHD1 to the cytoplasm may be an additional strategy used by HCMV to evade host antiviral restriction. Indeed, Cryo-IEM images confirmed the localization pT592-SAMHD1 to the cytoplasm after HCMV infection and revealed for the first time its association with viral particles and dense bodies. The latter are electron-dense non-lysosomal vesicles that bud from the viral assembly complex and the Golgi apparatus, and like infectious viral particles, are associated with many different viral glycoproteins [1,70].

Interestingly, immunoprecipitation assays using antibodies to SAMHD1 and pT592-SAMHD1 allowed us to identify the binding of five different HCMV proteins to SAMHD1. Although it should be noted that the relative binding affinity of two antibodies may differ significantly, our results indicated that phosphorylation modulated the interaction of SAMHD1 with several viral proteins, a unique observation. Future studies will allow us to assess how SAMHD1 phosphorylation modulates the binding of these five proteins and their possible involvement in favouring SAMHD1 interaction with viral particles.

Based on the conclusion from the recent studies with HCMV [16–18], together with the present results, it is our hypothesis that SAMHD1 becomes phosphorylated at pT592 after HCMV infection mainly by Cdk2 and pUL97, leading to a negative regulation of the antiviral activity, possibly through an alteration in the conformation of the enzyme from the tetrameric active form to the monomeric inactive form, an aspect however still highly debated [34,40,43–46,52–53]. Phosphorylation could then change contacts with viral proteins and direct the incorporation of SAMHD1 into viral particles and/or non-infectious dense bodies.

In conclusion, if and how post-translational modifications, particularly phosphorylation, influence the antiviral capacity of SAMHD1, how HCMV counteracts host restriction, and the precise mechanisms through which HCMV exploits SAMHD1 activity, represent important research issues to be further addressed in ongoing studies. The answers to these questions could potentially provide the biological rationale to develop new therapeutic strategies for mitigating the impact of this opportunistic virus.

## Materials and methods

### Cells and culture conditions

Primary human foreskin fibroblasts (HFFs), the adult retinal pigment epithelial cell line ARPE-19, the human embryo kidney 293T (HEK 293T), and the human acute monocytic leukemia THP-1 cells were purchased from the American Type Culture Collection. HFFs and HEK 293T cells were grown in DMEM containing 10% FCS, 2 mM glutamine, 1 mM sodium pyruvate, 100 U/ml penicillin and 100 μg/ml streptomycin sulfate (Gibco). To obtain quiescent HFFs, we seeded 0.8x10^6^ cells per 25-cm flask in DMEM with 10% FCS (confluent cells) or with 0.1% FCS (serum-starved cells) and cultured them for 4 days. HFFs were used at passages 10–20. THP-1 cells, cultured as non-adherent monocyte-like cells, were grown in RPMI medium supplemented as above, and differentiated into macrophage-like cells by addition of 100 ng/ml of phorbol myristate acetate (PMA; Sigma-Aldrich) for 3 days, and then infected. All data with THP-1 cells were based on PMA-differentiated cells. ARPE-19 cells were grown in a 1:1 mixture of DMEM and Ham’s F-12 medium containing 10% FCS, 15 mM HEPES, 2 mM glutamine, 1 mM sodium pyruvate, 100 U/ml penicillin and 100 ug/ml streptomycin sulfate. Human microvascular endothelial cells (HMVEC) (dermal origin, CC-2543) were obtained from Clonetics, and cultured in endothelial growth medium as previously described [71]. Cells were used at passages 4–15. All cells were maintained at 37° C in a 5% CO_2_ atmosphere.

### HCMV preparation and infection

HCMV AD169 strain (ATCC VR538) was prepared as previously described [71,72], and viral stocks used in the experiments contained ~2 x 10^7^ PFU/ml. Standard plaque assays were used to titrate viral stocks, as well as in different experiments to determine viral titers in the supernatants harvested from infected cells [71,72]. UVB-inactivated AD169 was prepared using a double pulse of UV-B light (1.2 J/cm^2^).

HCMV TR was kindly provided by Prof. Jay A. Nelson (Oregon Health and Science University, USA) and reconstitution was performed as previously described [72]. HCMV VR1814 was previously described [71,72]. Merlin strain was kindly provided by Drs. Klaus Hamprecht and Gerhard Jahn (University of Tubingen, Germany). HCMV clinical isolates were obtained from urines of infants affected by congenital HCMV infection, as previously described [73] (Ethical Approval n. 007816 obtained by the Research Ethics Committee of the University Hospital of Turin “A.O.U. Città della Salute e della Scienza Torino–A.O. Ordine Mauriziano–A.S.L. TO1”).

Cells were infected at 80–90% confluence at the indicated MOI in their respective culture medium, without FCS. After 3 h (AD169 and TR strains) or 5 h (VR-1814 strain) at 37°C, virus inoculum was replaced with fresh culture medium (day 0). Mock-infected control cells were exposed for the same amount of time to an equal volume of medium. At various days post-infection (dpi), cells were harvested and analyzed.

When the pUL97 HCMV kinase inhibitors Maribavir (MBV) or Gö6976 (Gö; Merck Calbiochem), or the Cdk inhibitors CGP74514A (CGP; Merck Millipore), RO-3306, KO-3861 and BMS-265246 (Sellekchem) were used, HFFs were treated, infected and cultured as described in figure legends. As controls, cells were not treated or treated with DMSO.

### Real-time PCR

Total RNA was extracted using TRI Reagent solution (Life Technologies), according to the manufacturer’s instructions and 1 μg of total RNA was used for cDNA synthesis in a reaction volume of 20 μl. Real-time PCR was performed using the ABI PRISM 7000 PCR cycler (Applied Biosystems). cDNAs were amplified in triplicate with primers for SAMHD1 (Hs.PT.49a.21502281) and GAPDH (Hs.PT.49a.2918858.g) using specific TaqMan gene expression assays (Integrated DNA Technologies). Relative expression of each gene versus GAPDH was calculated according to the 2^-ΔΔCt^ method.

### Antibodies

The following antibodies were used in immunoblot: anti-SAMHD1 rabbit polyclonal antibody (Ab) (Proteintech); anti-SAMHD1 rabbit polyclonal Ab specific for the phosphorylated T592 residue (ProSci); rabbit polyclonal anti-p85 subunit of PI3K (N-SH2 domain) and mouse monoclonal antibody (mAb) anti-IE1/IE2 viral proteins (MAB810R; Merck Millipore); mAb anti-UL97 (kindly provided by Thomas Mertens, Ulm University Medical Center, Germany); rabbit anti-Cdk1 (#77055) and mAb anti-tubulin (both from Cell Signaling); rabbit anti-Cdk2 (sc-163) and mAb anti-lamin A (C-3) (both from Santa Cruz). Horseradish peroxidase-conjugated secondary antibodies were from GE Healthcare. Alexa Fluor 488–conjugated mAb anti-IE1/IE2 viral proteins (MAB810X; Merck Millipore) or FITC-conjugated anti-Ki67 (SolA15; eBioscience) were used in flow cytometry. In type I IFN blocking experiments, a neutralizing anti-human IFN alpha/beta receptor chain 2 (CD118) mAb (MMHAR-2; Merck Millipore) was used at the concentration of 1 μg/ml, and freshly added at 2 dpi.

### Immunoblot analysis

Cells were lysed for 20 min on ice in a lysis buffer containing 0.2% Triton X-100, 0.3% NP40, 1 mM EDTA, 50 mM TrisHCl pH 7.6, 150 mM NaCl, protease and phosphatase inhibitors to obtain whole-cell protein extracts. For nuclear/cytoplasm immunoblotting, cytosolic proteins were obtained by incubating cell pellets for 4 min on ice in a hypotonic lysis buffer containing 0.5% NP-40, 50 mM KCl, 25 mM Hepes pH 7.8, DTT 100 μM, protease, and phosphatase inhibitors. Lysates were then centrifuged at 5000 rpm for 5 min, and supernatants containing cytoplasmic content stored at -80°C. The remaining nuclear pellets were resuspended in the nuclear extraction buffer containing 500 mM KCl, 25 mM Hepes pH 7.8, 10% glycerol, DTT 100 μM, protease, and phosphatase inhibitors. After vortexing, lysates were put on rotation for 2 h at +4°C and then stored at -80°C. Protein concentrations of total, cytosolic and nuclear extracts were determined by the BCA method (BioRad). Lysates (20–30 μg) were resolved by SDS-PAGE and transferred to nitrocellulose membranes (Amersham, GE Healthcare). Membranes were blocked with 5% milk blocking buffer and probed with the indicated Abs. Lamin-A and α-tubulin were stained to validate fractionation efficiency. Densitometric analysis was performed with ImageJ software.

### Immunofluorescence and FACS analysis

Uninfected and infected cells were harvested at the indicated time post-infection, and intracellular staining of IE1/IE2 antigens was performed by fixation in 1% formaldehyde, permeabilization with 70% ethanol and then incubation with Alexa Fluor 488-conjugated anti-IE mAb (MAB810X; Merck Millipore). Cells were acquired with a FACS Canto flow cytometer (BD Biosciences) and analyzed with FlowJo 10 software.

### Small interfering RNA

The small interfering RNA (siRNA) specific for SAMHD1 (sc-76442), for Cdk1 (sc-29252), Cdk2 (sc-29259) and the non-targeting siRNA (siCtrl) (sc-37007) were from Santa Cruz Biotechnology. Three different siRNAs (1: AF520960.1s, AF520960.1as; 2: AF520960.2s, AF520960.2as; 3: AF520960.5s, AF520960.5as) targeting the UL97 gene were purchased from Sigma-Aldrich. HFFs (90% confluence) were transfected with 100–300 nM siRNA using DharmaFECT siRNA transfection reagent (Thermo Fisher Scientific), according to the manufacturer’s recommendation. One or two days after transfection, cells were infected with AD169 at the indicated MOI, and then cells and supernatants were harvested and analyzed as indicated.

### Viral like particles (VLP) preparation

For VLP production, 3.5x10^6^ 293T cells were transiently transfected with SIV-based packaging plasmid with or without Vpx protein (pAdSiv3+or pAdSiv3+/ΔVpx respectively) and VSV.G plasmid, in a ratio 2:1 by Calcium Phosphate method, using the Profection Mammalian Transfection System (Promega Corporation, Madison, WI, USA) as previously described [74]. Two-three days post-transfection, cell culture supernatants were collected, cleared from cellular debris by low-speed centrifugation and passed through a 0.45-μm pore size filter (Millipore Corporation, Billerica, MA, USA). VLPs stocks were titred by the reverse transcriptase (RT) activity (1.1x10^6^ cpm/ml). For HFF challenge, VLPs at an MOI of 1 were adsorbed by spinoculation at 1500 rpm for 30 min at room temperature, then incubated for 2 h at 37° C in a 5% CO_2_ atmosphere, and soon after the same cells were infected with AD169 at an MOI of 1. At different days post-infection, cells and supernatants were harvested and subjected to immunoblotting and plaque assays, respectively.

### Confocal microscopy analysis

To stain intracellular IE and SAMHD1 (total and phosphorylated), HFFs were starved in 0.2% FCS for 48 h, and then infected with AD169 at an MOI of 1. At 2 dpi, cells were harvested and re-plated on PBS 2% gelatin-coated multichamber slides for additional 24 h. Cells were then fixed with PBS 4% paraformaldehyde, permeabilized with PBS 0.1% Triton X-100, and then blocked with PBS 0.01% Triton X-100 and 10% human serum obtained from a HCMV seronegative donor. Cells were then incubated with the indicated primary antibodies for 1 h at R/T, washed and incubated with Alexa-Fluor 488-conjugated goat anti-mouse and Alexa-Fluor 594-conjugated goat anti-rabbit secondary antibodies (ThermoFisher), for 1 h at R/T. All incubations with antibodies were performed in the presence of 5% human HCMV seronegative serum. Cells were then washed and counterstained with DAPI (D1306; Life Technologies). Coverslips were mounted using SlowFade gold reagent (S36936; Life Technologies). Fluorescence images were acquired with IX83 FV1200 MPE laser-scanning confocal microscope using a 60 × /1.35 NA UPlanSAPO oil immersion objective (all from Olympus). Sequential acquisition was used to avoid crosstalk between different fluorophores. Images were acquired with FluoView 4.2 software and processed with Fiji/ImageJ software. Fluorescence intensity (F.I.) quantification was performed with Fiji/ImageJ upon background subtraction. DAPI staining was used as a segmentation mask to separate the nucleus from the cytoplasm and to select a region of interest (ROI) in which nuclear red signal intensity (SAMHD1 or p-T592 SAMHD1) was measured. Cytoplasmic-localized fluorescence was then calculated by subtracting nuclear signal from the total cellular fluorescence.

### Cryo-immunoelectron microscopy

Cells grown in monolayer were fixed in 2% PFA and 0.2% glutaraldehyde in 0.1 M PBS, pH 7.4, for 3 h at room temperature, then embedded into 12% gelatin in 0.1 M PBS, pH 7.4, solidified on ice, infused in 2.3 M sucrose overnight at 4°C, mounted on aluminum pins and frozen in liquid nitrogen. Ultrathin cryosections were cut at -110°C using an Ultracut EM FC6 (Leica Microsystems, Vienna, Austria), collected with 1% methylcellulose in 1.15 M sucrose and indirectly single-labeled. Samples were first blocked with 10% human serum obtained from a HCMV seronegative donor diluted in PBS, and then immunolabeled with primary antibodies to SAMHD1 (Proteintech) and phospho-SAMHD1 (ProSci). Bound antibodies were visualized using goat anti-rabbit conjugated with 5-nm (Sigma) or 10-nm gold particles (Cytodiagnostics). All incubations with antibodies were performed in the presence of 2% human HCMV seronegative serum. Cryosections were analyzed with a Philips CM10 TEM and Fei-Philips Morgagni 268D) [75].

### Antibody pull-down followed by LC-MS/MS

Protein lysate from HFFs infected with AD169 at an MOI 1 and MOI 3 at 3 dpi were prepared and a total of 2 mg of cell lysate was used for immunocapture followed by LC-MS/MS. Approximately 45 μg of protein was loaded, in triplicates, on a precast 4–20% Criterion TGX StainFree Gel (BioRad Laboratories), together with PageRuler Plus Prestained Protein Ladder (Thermo Scientific), and run according to manufacturer’s recommendation. The gel was fixed in 25% isopropanol and 10% acetic acid for 10 min, stained using in 10% acetic acid and 0.006% Coomassie brilliant blue G-250 for 30 min, and destained through washing in 10% acetic acid for 16 h. The full lysate was excised from the gel and in gel digestion was performed as previously described [76] with some changes to the original protocol as outlined below. Briefly, gel pieces were shrunk in 100% Acetonitrile (ACN) and reduced by 10 mM DTT for 30 minutes at 56°C. Alkylation was performed by addition of 55 mM 2-choloroacetamide and incubated in dark for 20 minutes. Trypsin digestion was performed over night at 37°C after addition of trypsin (13 ng/μl in 50 mM ammonium bicarbonate, 10% ACN (vol/vol)). Peptides were extracted by addition of 100 μl of extraction buffer (1:2 (vol/vol) 5% formic acid/ACN) and dried down by vacuum centrifugation prior to LC-MS/MS analysis. Each sample was analyzed on a HF Q-Exactive Orbitrap (Thermo Fisher) connected to a Dionex UHPLC system (Thermo Fisher Scientific). The UHPLC was equipped with a trap column (Acclaim PepMap 100, 75 μm x 2 cm, nanoviper, C_18_, 3 μm, 100 Å; Thermo Fisher Scientific) and an analytical column (PepMap RSLC C_18_, 2 μm, 100 Å, 75 μm x 25 cm; Thermo Fisher Scientific). Mobile-phase buffers for nLC separation consisted of 0.1% Formic Acid (FA) in water (solvent A) and 80% ACN/0.1% FA (solvent B). The peptides were eluted during a 60-minute gradient and directly sprayed into the mass spectrometer. The flow rate was set at 250 nl/min, and the LC gradient was as follows: 5% solvent B for 3 min, 5–35% solvent B within 60 min, 35–90% solvent B within 5 min, 90% solvent B within 10 min and 5% solvent B for 10 min. Nano spray was achieved with an applied voltage of 1.8 kV. The mass spectrometer was programmed in a data-dependent acquisition mode (top 10 most intense peaks) and was configured to perform a Fourier transform survey scan from 370 to 1,600 *m/z* (resolution 60,000), AGC target 3e^6^, maximum injection time 250 ms. MS2 scans were acquired on the 10 most-abundant MS1 ions of charge state 2–7 using a Quadrupole isolation window of 1 m/z for HCD fragmentation. Collision energy was set at 34%; resolution = 30 000; AGC target 2 e^5^, maximum injection time 200 ms; dynamic exclusion 15 s. Raw files for the Intensities for label free-MS quantification was performed by analyzing the raw data by MaxQuant (version 1.5.7.0). Andromeda was used to search the MS/MS data against the Uniprot *Homo sapiens* database (42,259 entries downloaded 20180115) complemented with a list of common contaminants and concatenated with the reversed version of all sequences. Trypsin/P was chosen as cleavage specificity allowing two missed cleavages. Carbamidomethylation (C) was set as a fixed modification, while oxidation (M) was used as variable modification. Data filtering was carried out using the following parameters: peptide and protein FDRs were set to 1%, minimum peptide length was set to 7. The reverse and common contaminant hits were removed from MaxQuant. Proteins intensities from MaxQuant were compared between the SAMHD1 enriched proteome versus the negative control (pooled Rabbit IgG). For the fold change (FC) analysis between anti-SAMHD1 and anti-pT592-SAMHD1 pull-downs, cell lysates from HFFs not infected or infected with AD169 (MOI 1 and MOI 3, 3 dpi) were immunoprecipitated by protein A Sepharose beads complexed with the specific anti-SAMHD1 Ab, or rabbit IgG as a control. HCMV proteins with FC > 1 and p < 0.05 (p-value Bonferroni corrected) were considered as differentially expressed. The Protein Group intensities per replicate were used as proxy for protein levels (label free quantification) and compared across different experimental conditions.

### Statistical analysis

Statistical analysis of the data was performed using a paired Student *t* test. A *p* value <0.05 was considered significant.

### Ethics statement

The study was approved by the Research Ethics Committee of the University Hospital of Turin “A.O.U. Città della Salute e della Scienza di Torino–A.O. Ordine Mauriziano–A.S.L. TO1” (No. 007816). Informed consent was obtained from parents of all study participants prior to collection of clinical data and biological samples. The study was carried out in accordance with the Declaration of Helsinki.

## Supporting information

S1 Fig Effect of type I IFN blocking on SAMHD1 expression. HFFs were infected at an MOI 1 or not infected (n.i.) in the presence of a neutralizing anti-human IFN alpha/beta receptor chain 2 (CD118) (IFNR) mAb, or an isotype control mAb (cIgG), both at the concentration of 1 μg/ml. Cells were harvested at 1 dpi or 3 dpi and, in the latter condition, new mAb was added at 2 dpi. a) Real-time PCR at 1 dpi was performed using primers specific for SAMHD1, or for the housekeeping gene GAPDH. Data from two independent experiments, expressed as fold change units ± SE, were normalized with GAPDH and referred to n.i./cIgG cells considered as calibrators and set at 1. *, p < 0.05. b) SAMHD1 levels were analyzed by immunoblotting in cell lysates of HFFs treated as described above. Expression of IE1/IE2 viral antigens was used as control for infection, while the p85 subunit of PI3K was used as loading control. A representative experiment out of two is shown. c) The relative amount of SAMHD1 protein, normalized to that of p85, was determined by densitometric analysis and is relative to that of n.i./cIgG cells, which was arbitrarily set as 1. Data are expressed as mean ± SE of two independent experiments. No statistically significant difference was observed in any combination.
(TIF)

S2 Fig SAMHD1 siRNA decreases both anti-SAMHD1 and anti-pT592-SAMHD1 staining. HFFs were transfected with SAMHD1 siRNA or non-targeting siRNA (ctrl). Two days later, cells were infected with AD169 at an MOI of 1 for 3 days. Stainings were then performed using primary antibodies directed against total (b and e) or pT592-SAMHD1 (i and l), followed by Alexa Fluor 594-conjugated goat anti-rabbit (red). Nuclei were stained with DAPI (a, d, h, k) (blue). Confocal images are shown as single optical slice from one representative experiment out of two. An overlay of blue/red images (c, f, j, m) is also shown. Scale bar: 10μm. Red fluorescence intensity was measured with Fiji/ImageJ software in 70 (g) or 150 (n) cells randomly acquired from two independent experiments. Histograms represent the mean ± SE. * p < 0.05; *** p < 0.001.
(TIF)

S3 Fig Volcano plot showing LC-MS/MS data from affinity pull-down experiments. Cell lysates from HFFs not infected or infected with AD169 (MOI 1 and MOI 3, 3 dpi) were immunoprecipitated with anti-SAMHD1 or anti-pT592-SAMHD1 Ab, or rabbit IgG as a control. Points indicate identified proteins from each experimental condition using the two antibodies. Significantly enriched genes (p < 0.01) are labelled, with human genes (in gray) and HCMV genes (in blue) visualized. a) Difference between SAMHD1 at MOI 1 and MOI 3. b) Difference between pSAMHD1 at MOI 1 and MOI 3. c) Difference between SAMHD1 and pT592-SAMHD1 at MOI 1. d) Difference between SAMHD1 and pSAMHD1 at MOI 3.
(TIF)

S4 Fig Excel spreadsheet containing, in separate sheets, the underlying numerical data for Figure panels 1A, 1B, 2B, 2E, 3B, 3D, 3E, 3G, 3H, 3J, 4D, 5B, 5C, 5D, 6B, 6C, 7I, 7J, 7S, 7T, 8B and 8C, Tables 1 and 2, S1A, S1C, S2G and S2N Figs. (XLSX)
