# Supplementary figures and images for: SAMHD1 phosphorylation and cytoplasmic relocalization after human cytomegalovirus infection limits its antiviral activity
Source: PLoS Pathog. 2020 Sep 28;16(9):e1008855. doi: 10.1371/journal.ppat.1008855 (PMC7544099; doi:10.1371/journal.ppat.1008855)

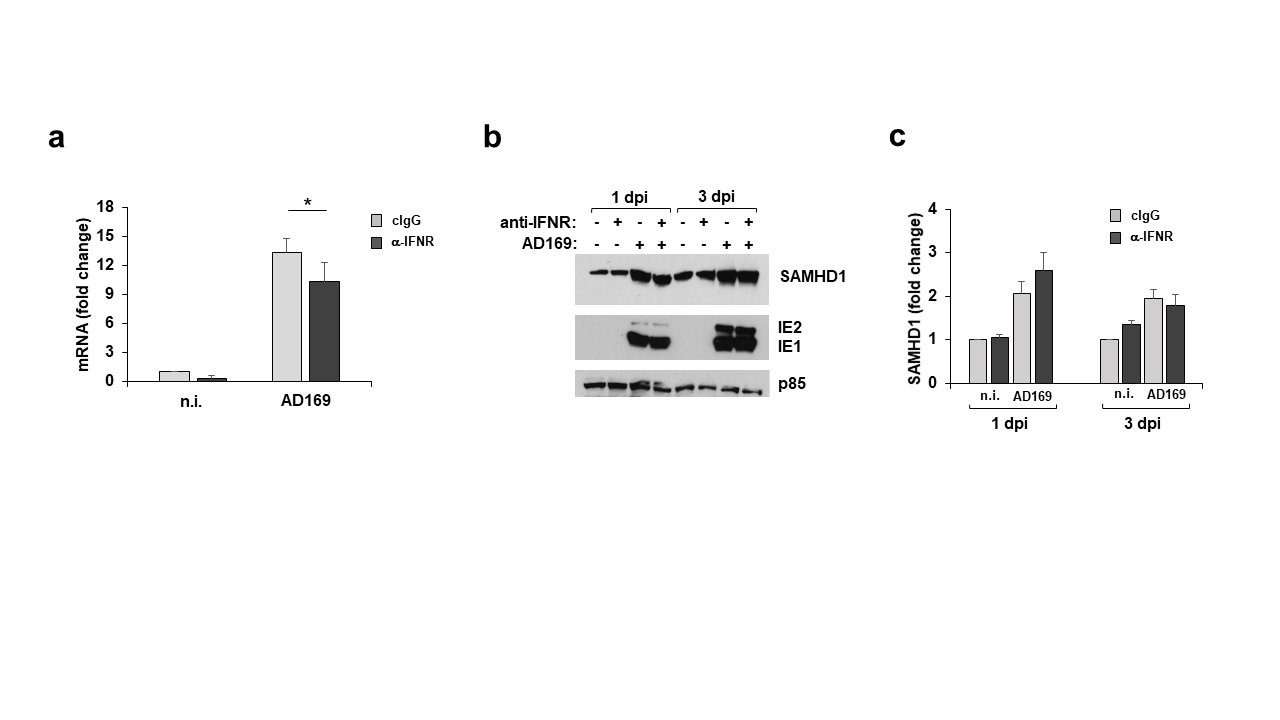

Supplement: S1 Fig — HFFs were infected at an MOI 1 or not infected (n.i.) in the presence of a neutralizing anti-human IFN alpha/beta receptor chain 2 (CD118) (IFNR) mAb, or an isotype control mAb (cIgG), both at the concentration of 1 μg/ml. Cells were harvested at 1 dpi or 3 dpi and, in the latter condition, new mAb was added at 2 dpi. a) Real-time PCR at 1 dpi was performed using primers specific for SAMHD1, or for the housekeeping gene GAPDH. Data from two independent experiments, expressed as fold change units ± SE, were normalized with GAPDH and referred to n.i./cIgG cells considered as calibrators and set at 1. *, p < 0.05. b) SAMHD1 levels were analyzed by immunoblotting in cell lysates of HFFs treated as described above. Expression of IE1/IE2 viral antigens was used as control for infection, while the p85 subunit of PI3K was used as loading control. A representative experiment out of two is shown. c) The relative amount of SAMHD1 protein, normalized to that of p85, was determined by densitometric analysis and is relative to that of n.i./cIgG cells, which was arbitrarily set as 1. Data are expressed as mean ± SE of two independent experiments. No statistically significant difference was observed in any combination. (TIF) [file ppat.1008855.s001.tif]

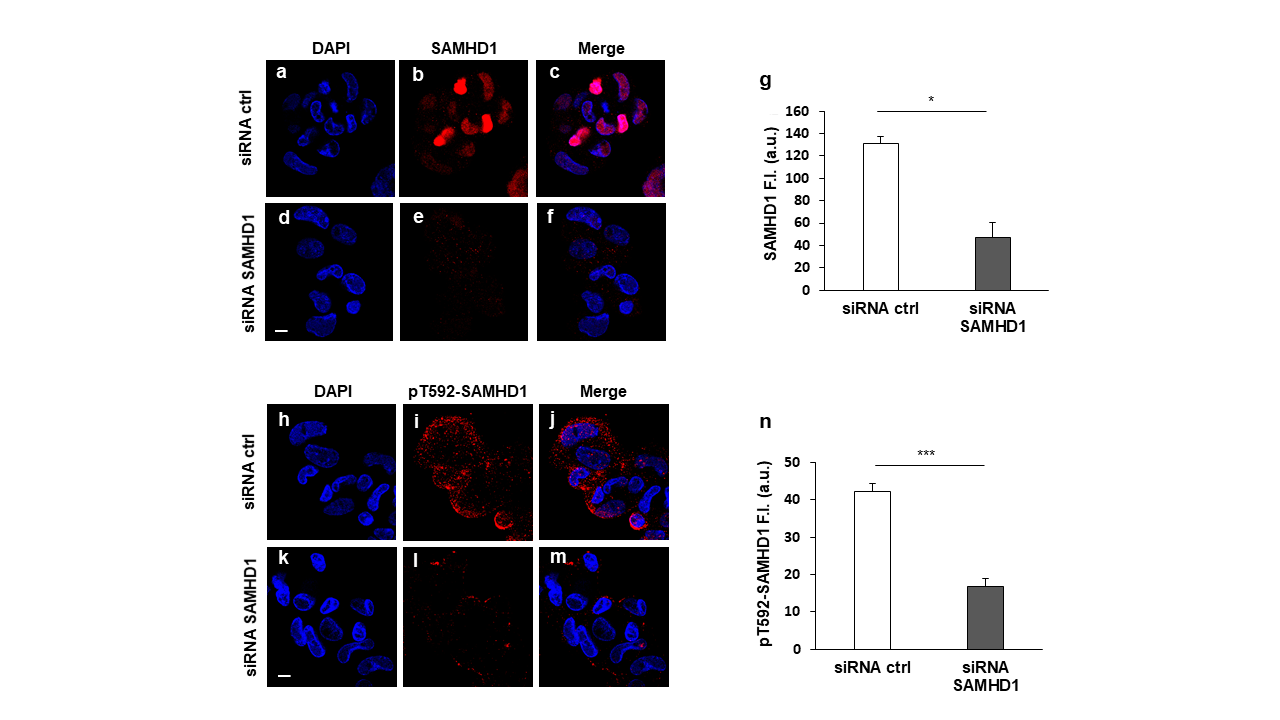

Supplement: S2 Fig — HFFs were transfected with SAMHD1 siRNA or non-targeting siRNA (ctrl). Two days later, cells were infected with AD169 at an MOI of 1 for 3 days. Stainings were then performed using primary antibodies directed against total (b and e) or pT592-SAMHD1 (i and l), followed by Alexa Fluor 594-conjugated goat anti-rabbit (red). Nuclei were stained with DAPI (a, d, h, k) (blue). Confocal images are shown as single optical slice from one representative experiment out of two. An overlay of blue/red images (c, f, j, m) is also shown. Scale bar: 10μm. Red fluorescence intensity was measured with Fiji/ImageJ software in 70 (g) or 150 (n) cells randomly acquired from two independent experiments. Histograms represent the mean ± SE. * p < 0.05; *** p < 0.001. (TIF) [file ppat.1008855.s002.tif]

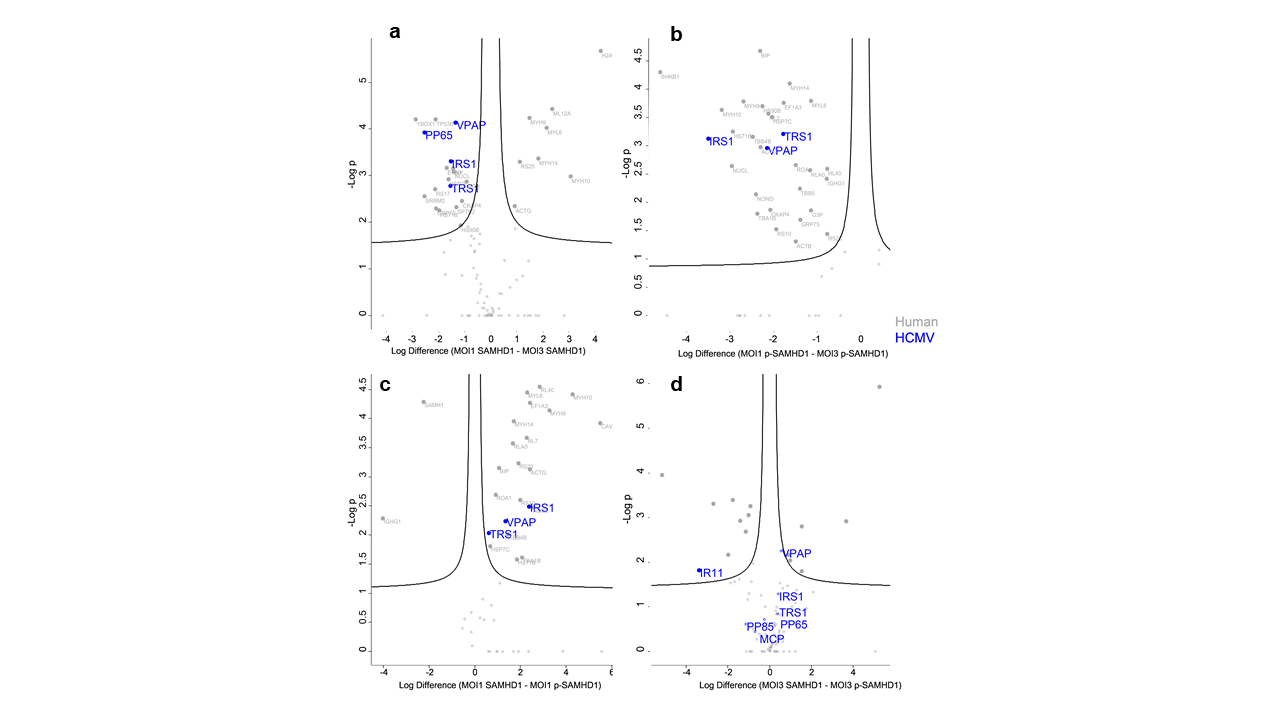

Supplement: S3 Fig — Cell lysates from HFFs not infected or infected with AD169 (MOI 1 and MOI 3, 3 dpi) were immunoprecipitated with anti-SAMHD1 or anti-pT592-SAMHD1 Ab, or rabbit IgG as a control. Points indicate identified proteins from each experimental condition using the two antibodies. Significantly enriched genes (p < 0.01) are labelled, with human genes (in gray) and HCMV genes (in blue) visualized. a) Difference between SAMHD1 at MOI 1 and MOI 3. b) Difference between pSAMHD1 at MOI 1 and MOI 3. c) Difference between SAMHD1 and pT592-SAMHD1 at MOI 1. d) Difference between SAMHD1 and pSAMHD1 at MOI 3. (TIF) [file ppat.1008855.s003.tif]
